# Clara Barton

**Published:** 1912-05

**Authors:** 


So many elaborate biographies and eloquent tributes have been written to the memory of the late Clara Barton, that we need only mention the death of this venerable heroine of two wars, who has done so much to secure for the medical profession the skilled assistance of defter hands and more intuitive minds than belong to man. All honor to her memory.

